# Evaluation of thromboelastometry parameters as predictive markers for sinusoidal obstruction syndrome in patients undergoing allogeneic stem cell transplantation for acute leukaemia

**DOI:** 10.18632/oncotarget.18499

**Published:** 2017-06-15

**Authors:** Joanna Rupa-Matysek, Lidia Gil, Ewelina Wojtasińska, Zuzanna Kanduła, Adam Nowicki, Magdalena Matuszak, Mieczysław Komarnicki

**Affiliations:** ^1^ Department of Hematology and Bone Marrow Transplantation, Poznan University of Medical Sciences, Poznań, Poland

**Keywords:** sinusoidal obstruction syndrome, acute leukemia, allogeneic stem cell transplantation, biomarkers, thromboelastometry

## Abstract

Hepatic sinusoidal obstruction syndrome (previously named veno-occlusive disease, SOS/VOD) is a serious complication of allogeneic stem cell transplantation (HSCT). Early identification of patients at risk of SOS/VOD may possibly improve the outcome and reduce mortality. Rotation thromboelastometry (ROTEM) is global assay allowing for the precise assessment of both bleeding and thrombotic conditions, however, its usefulness in patients undergoing HSCT for acute leukaemia has not been studied.

We evaluated the thromboelastometry parameters in patients undergoing allogeneic HSCT for acute leukaemia to identify candidate biomarkers of SOS/VOD occurrence.

ROTEM assays (INTEM, EXTEM, FIBTEM, APTEM) were performed on day -10, on the day of stem cell infusion (day 0) and on days +12 and +28 post-HSCT. The diagnosis of SOS/VOD was based on the Baltimore criteria. Seven patients (26%) developed SOS/VOD. On day +12, the patients with SOS/VOD had statistically significant longer INTEM-CT (clotting time, 199 ± 33.41vs166 ± 23.65s, *p* = 0.0033), EXTEM-CT (69.5 ± 6.39vs.52 ± 3.42s, *p* = 0.0139) and FIBTEM-CT (69.5 ± 22.75vs. 50.8 ± 14.31s, *p* = 0.0124) compared to SOS/VOD (-). ROC curve on day +12 indicated a cut-off value of 179s in INTEM-CT (AUC = 0.91), 69s in EXTEM-CT (AUC = 0.90) and 102s in FIBTEM-CT (AUC = 0.82) for the prediction of SOS/VOD.

This is the first study evaluating the usefulness of ROTEM assays in the early detection of haemostasis abnormalities predictive of SOS/VOD development in patients undergoing HSCT for acute leukemia. Patients with SOS/VOD had a significant delay in the initiation of thrombin formation in the analysed ROTEM assays. The utility of ROTEM assays in the optimal management of patients undergoing HSCT should be clarified in further prospective studies.

## INTRODUCTION

Sinusoidal obstruction syndrome, previously called veno-occlusive disease of the liver (SOS/VOD), is a serious complication after haematopoietic stem cell transplantation (HSCT). SOS/VOD is more common after allogeneic HSCT (allo-HSCT) conditioned with a myeloablative regimen (MA), where it has an incidence of around 10–15% (ranging up to 40% in some studies), against < 5% after a reduced intensity conditioning regimen (RIC) and autologous HSCT [1, 2]. The diagnosis of SOS/VOD is based on clinical criteria including jaundice, painful hepatomegaly or ascites, and/or unexplained weight gain in a patient who fulfils either the modified Seattle criteria or the Baltimore criteria [3, 4]. Despite the fact that the incidence rate of SOS/VOD is relatively low and most cases of mild SOS/VOD are self-limiting, a severe syndrome with multi-organ failure is still associated with a high mortality rate, reaching 80% [1, 2, 5]. Therefore, early diagnosis and early treatment is essential to prevent disease progression to severe SOS/VOD and may possibly reduce mortality. The risk factors associated with increased risk of the development of SOS/VOD include patient-related factors such as age, underlying disease, liver damage, iron overload and previous/concomitant hepatotoxic drugs; and transplant-related factors; number of HSCT, type of transplant and donor, and the type of conditioning regimen the patient receives [5–7]. Preliminary data suggest that for early and accurate diagnosis of SOS, several potential biomarkers, or a panel of biological markers including those of endothelial injury or haemostatic parameters involved in SOS/VOD pathogenesis (especially plasminogen activator inhibitor-I, PAI-1), may be of some interest [5, 8].

The pathogenesis of SOS/VOD is complex and includes damage to endothelial cells and hepatocytes in zone 3 of the liver acinus. These are caused by toxic metabolites generated by the conditioning regimen, along with the cytokines released by the damaged tissues. All these processes lead to post-sinusoidal hypertension and the hepatorenal syndrome [5, 9]. Changes in activity of coagulation factors due to endothelial cell injury and activation of fibrinolytic pathways not only contribute to SOS/VOD pathogenesis but may also be used to predict the occurrence of SOS/VOD [10, 11]. A hypercoagulable state during HSCT, profound thrombocytopenia and intravenous catheter all predispose to haemostasis abnormalities [12].

Thromboelastometry provides viscoelastic testing of haemostasis in whole blood and allows for the simultaneous evaluation of the different components involved in clot formation; plasma factors with contact activation and tissue factor activation, the contribution of platelets, fibrinogen and fibrinolysis [13].

The aim of the present study was to assess the coagulation abnormalities in patients treated for acute leukaemia with allogeneic HSCT by rotation thromboelastometry and to identify coagulopathy with respect to the development of SOS/VOD. The INTEM, EXTEM, FIBTEM and APTEM assays were all investigated.

## RESULTS

### Patient characteristics

This prospective observational study enrolled 27 adult patients, 14 males and 13 females. Median patient age was 38 years (range 18–63). Underlying diseases included acute myeloid leukaemia (AML, *n* = 20) or acute lymphoblastic leukaemia (ALL, *n* = 7). The patients underwent allogeneic stem cell transplantation from HLA-matched unrelated donors (*n* = 22) or sibling donors (*n* = 5) after myeloablative (*n* = 16) or reduced-intensity conditioning regimen (*n* = 11). Allogeneic peripheral blood stem cells (*n* = 16, 59%), bone marrow (*n* = 10, 37%), or cord blood (*n* = 1) were used as stem cell sources. The patients demographic and transplant characteristics are presented in Table 1.

**Table 1:** Clinical characteristics of patients and grafts

Parameter	*n*
**Number of patients**	27
**Gender**, female/male	13/14
**Age**; median, range years	38 (18–63)
**Diagnosis/Disease**	
AML	20
ALL	7
**Donor type**	
● sibling	5
● unrelated	22
**Stem cell source**	
● peripheral blood	16
● bone marrow	10
● cord blood	1
**Conditioning regimens**	
● myeloablative (MA)	16
BuCy	9
TBICy	7
● reduced-intensity (RIC)	11
Fludarabine-based regimen	11
FluMel	6
FluBu2	5
**GvHD prophylaxis**	
● MTX+CsA	5
● MTX+CsA+ATG	22
**Other risk factors**	
Second HSCT	7
Advanced disease (beyond second CR or relapse)	5
Iron overload (ferritin > 1000 ng/ml)	7
Previous liver disease	2
**Complications**	
● Acute GvHD	6
● Chronic GvHD	3
● SOS/VOD	7
● Gram negative bloodstream infections	4
● Central venous catheter-related thrombosis	1
● Serious bleeding event	0
● Organ toxicity grade 3 or 4	0

Abbreviations: AML - acute myeloid leukaemia, ALL - acute lymphoblastic leukaemia, ATG – anti-thymocyte globulin, BuCy - busulfan plus cyclophosphamide, CsA – ciclosporin, FluMel – fludarabine plus melphalan, GvHD - graft versus host disease, MAC - myeloablative conditioning regimen, MTX – methotrexate, RIC - reduced-intensity conditioning regimen, SOS/VOD - sinusoidal obstraction syndrome, TBI Cy - total body irradiation plus cyclophosphamide.

All the patients engrafted in a median time of 21 days (range 15–29 days). Acute graft versus host disease (GvHD) was diagnosed in 22% of patients, while chronic GvHD developed in 11%. In all the patients who developed acute graft versus host disease (aGvHD), the symptoms occurred 30 days post-HSCT.

### SOS/VOD

All analysed patients had two or more risk factors for developing SOS/VOD according to published data and were considered as high risk. Seven patients developed SOS/VOD (26%). Mild SOS/VOD occurred in 5 (71%) patients and moderate in 2 (29%) patients. None of them had a severe SOS/VOD or developed multiple organ failure. None of the patients who developed SOS/VOD died. The median time to SOS/VOD diagnosis after HSCT was 13 days (range 12–16). Among the aGVHD group, there was only one patient who developed mild SOS/VOD with the disappearance of all SOS/VOD symptoms before the onset of aGVHD. Weight gain, jaundice and hepatomegaly were the most common manifestations. Therefore, the treatment was based on fluid restriction and diuretics and led to resolution of symptoms in all patients.

The patients with or without, symptoms of SOS/VOD did not differ with regard to age, gender, type of acute leukaemia (AML versus ALL), type of donor (unrelated versus sibling), stem cell source (peripheral blood versus bone marrow stem cell) and intensity of conditioning. In those who received myeloablative regimen, no statistically significant differences were found between the patients conditioned with busulfan or TBI, Table 2.

**Table 2:** Clinical characteristics of the SOS/VOD positive and negative patients

Risk factors	SOS/VOD (+)	SOS/VOD (−)	*p* value
	*n* = 7	*n* = 20	
**Transplant-related factors**			
Myeloablative conditioning (TBICy+BuCy)	4 (57%)	12 (60%)	0.8946
BuCy	1 (14%)	8 (40%)	0.2142
TBICy	3 (43%)	4 (20%)	0.2349
Unrelated donor	7 (100%)	15 (75%)	0.1427
Second HSCT	1 (14%)	6 (30%)	0.4142
**Patient- and disease-related factors**			
Age > median 38 years	3 (43%)	11 (55%)	0.5800
Female gender	2 (28%)	11 (55%)	0.2284
AML	4 (57%)	16 (80%)	0.2349
Advanced disease (beyond second CR or relapse)	2 (29%)	3 (15%)	0.4263
Iron overload (ferritin > 1000 ng/ml)	6 (75%)	1 (5%)	0.0247
Previous liver disease	1 (14%)	1 (5%)	0.4195

Abbreviations: BuCy - busulfan plus cyclophosphamide, TBI Cy - total body irradiation plus cyclophosphamide.

*p* value between SOS (+)/VOD and SOS/VOD (−) group > 0.05 – not significant.

*p* value < 0.05 – statistically significant.

The median ferritin level was significantly higher in the patients who developed SOS (3218, range 1352–4970 ng/ml) in comparison to the patients who did not (598, range 152–3625 ng/ml; *p* = 0.0239). Consistent with recent literature, our data show that a pre-transplant ferritin level above 1000 ng/dl was associated with SOS/VOD development (*p* = 0.0247), Table 2.

### Coagulation parameters in the SOS/VOD patients

No statistically significant differences in the APTT and the PT on day +12, between the group with SOS/VOD symptoms (SOS/VOD+) and the group without SOS/VOD were found. Although the SOS/VOD (+) group had a higher median plasma fibrinogen concentration, no marked differences in the median fibrinogen level were observed at the time of SOS/VOD occurrence between the SOS/VOD (+) and SOS/VOD (−) groups. As in all HSCT recipients, the platelet count was below the reference range on day +12 and no significant reductions in the platelet count were seen in the patients with symptoms of SOS/VOD, Table 3.

**Table 3:** Platelet and plasma coagulation laboratory results from HSCT recipients with or without SOS/VOD during the study on day +12 after HSCT

Parameter	SOS/VOD (+)	SOS/VOD (−)	*p* value
	ME ± SE (Q1; Q3)		
APTT [s]	34.4 ± 3.62 (33.3–43.8)	34.3 ± 8.22 (30.85–45.15)	0.973
PT [s]	16.2 ± 2.34 (13.1–15.2)	14.25 ± 4.69 (12.7–15.15)	0.734
Platelet count [× 10^9^/l]	22.0 ± 2.70 (20.0–29.0)	22.66 ± 4.70 (16.8–34.55)	0.786
Fibrinogen [g/l]	4.55 ± 0.36 (4.19–4.92)	3.14 ± 0.25 (2.37–4.16)	0.106

Abbreviations: APTT – activated partial tromboplastin time [s]; PT – prothrombin time [s].

Data are expressed as median ± standard error (interquartile range) - Me ± SE (Q1;Q3).

*p* value > 0.05 – not significant.

### ROTEM parameters in the SOS/VOD group

#### Parameters concerning initiation and speed at which a solid clot forms (CT, CFT, α-angle)

**Figure 1:**
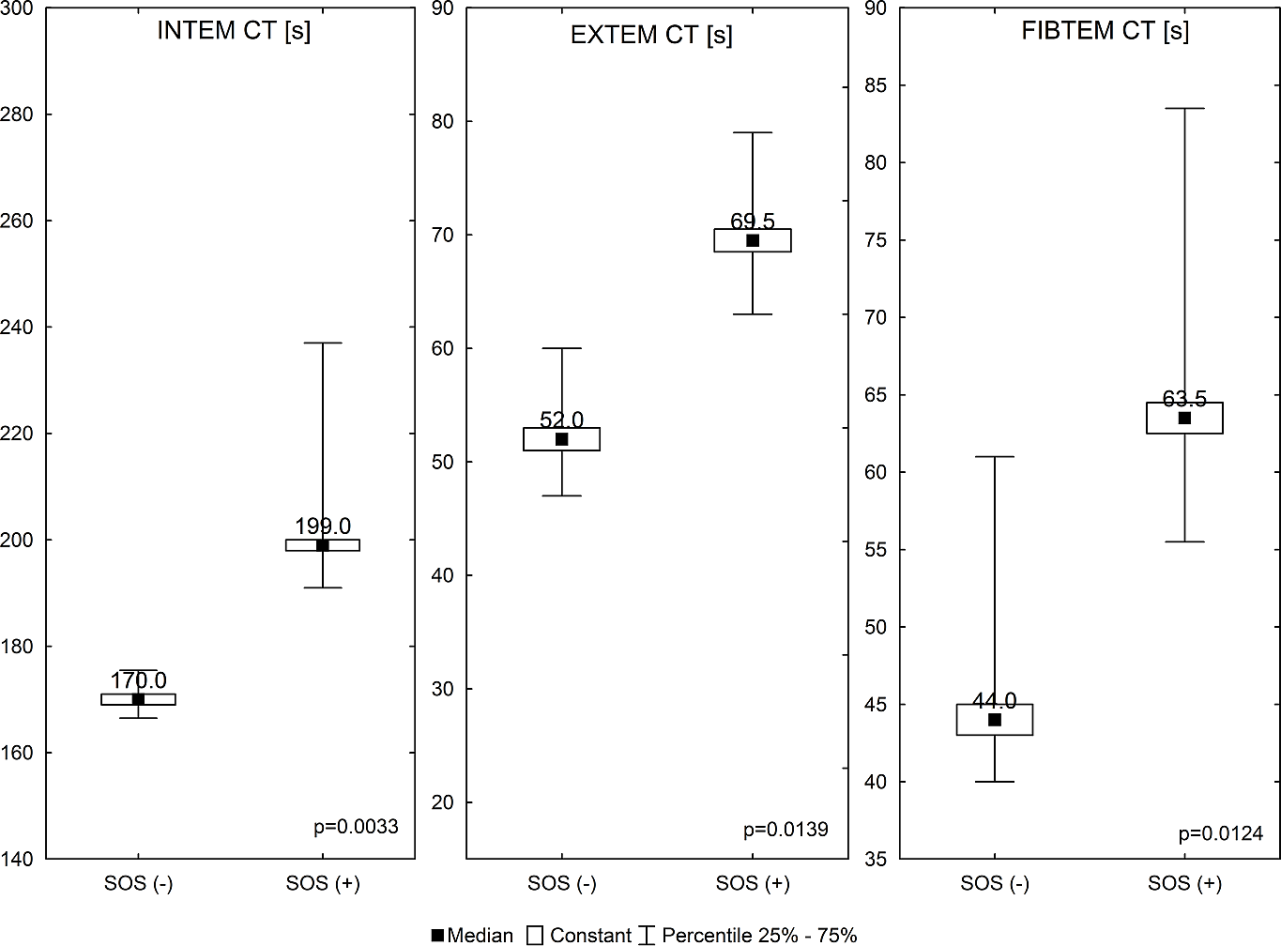
Comparison of selected ROTEM parameters between the SOS/VOD (+) group and SOS/VOD (−) group Abbreviations: CT – clotting time [s].

Compared with patients without SOS/VOD, the SOS/VOD (+) group had significantly longer times between the activator being added and initial clot formation (coagulation time, CT) on day +12 in the INTEM (Me ± SE, 199 ± 33.41 versus 166 ± 23.65 s, *p* = 0.0033), EXTEM (69.5 ± 6.39 versus 52 ± 3.42 s, *p* = 0.0139) and FIBTEM (69.5 ± 22.7 versus 50.8 ± 14.31 s, *p* = 0.0124) assays, Figure 1.

In the SOS/VOD (+) group, no significant differences were found in the clot formation time (CFT), defined as the interval between the onset of coagulation and the clot reaching an amplitude of 20 mm, and the values of α-angle, referring to the steepness of the curves, in comparison to the patients without SOS/VOD. The thromboelastometry values are shown in Table 4.

**Table 4:** Comparison of standard ROTEM profiles between the SOS/VOD (+) group and the SOS/VOD (−) group

ROTEM data		SOS/VOD (+)	SOS/VOD (-)	*p* value	SOS/VOD (+)	SOS/VOD (-)	*p* value
	Me ± SE (Q1; Q3)					Me ± SE (Q1; Q3)	
	day +12 after transplantation				day +28 after transplantation		
**INTEM**	**CT**	199 ± 33.41 (191; 237)	166 ± 23.65 (166; 175)	0.0033	193.5 ± 17.82 (178; 224.5)	173 ± 6.94 (160; 185)	0.2373
	**CFT**	110 ± 30.91 (71.5; 165)	131 ± 22.36 (73; 203)	0.5304	162.5 ± 53.56 (132.5; 275)	82 ± 25.03 (72; 144)	0.1199
	**Alfa angle**	78.5 ± 0.65 (77.5; 79.5)	78 ± 0.73 (75; 80)	0.6646	73 ± 6.07 (63; 78.5)	76 ± 0.76 (74; 78)	0.3168
	**A10**	35.5 ± 2.95 (32; 40.5)	35 ± 1.91 (29; 40)	0.6646	29.5 ± 2.56 (26.5; 34)	51 ± 2.96 (36; 54)	0.0404
	**A15**	39 ± 2.32 (36.5; 43)	38 ± 1.92 (32; 42)	0.4107	33.5 ± 2.9 (30; 39)	55 ± 3.08 (41; 59)	0.0404
	**A20**	42 ± 2.48 (38.5; 45.5)	40 ± 1.83 (35; 45)	0.4107	35.5 ± 2.75 (32.5; 41)	57 ± 2.83 (43; 59)	0.0518
	**A25**	43 ± 2.48 (39.5; 46.5)	42 ± 1.81 (37; 46)	0.5304	37 ± 3.01 (33.5; 43)	58 ± 2.72 (45; 60)	0.0518
	**MCF**	43.5 ± 2.46 (40.5; 47)	45 ± 4.24 (38; 52)	0.8096	41 ± 2.39 (39; 45.5)	58 ± 2.63 (46; 60)	0.0805
	**ML**	12 ± 0.57 (11; 12)	12 ± 0.22 (12; 12)	0.5067	10 ± 0.86 (8; 11)	9.5 ± 0.61 (7.5; 11.5)	0.9559
**EXTEM**	**CT**	69.5 ± 6.39 (63; 79)	52 ± 3.42 (47; 60)	0.0139	56 ± 6.56 (52; 68)	49 ± 2.49 (46; 50)	0.0805
	**CFT**	105 ± 26.76 (70.5; 143.5)	141 ± 24.06 (79; 213)	0.4107	132 ± 64.3 (117.5; 262.5)	94 ± 23.2 (64; 162)	0.2373
	**Alfa angle**	80 ± 0.75 (78.5; 81)	79 ± 0.79 (76; 81)	0.4690	79 ± 7.18 (65; 80)	77 ± 1.18 (73; 81)	0.8291
	**A10**	36.5 ± 2.06 (34; 39.5)	35 ± 1.97 (32; 40)	0.8096	33 ± 3.49 (27.5; 38.5)	52 ± 2.95 (38; 56)	0.0648
	**A15**	39.5 ± 1.87 (37.5; 42.5)	38 ± 1.91 (35; 44)	0.5965	37 ± 3.65 (31; 43)	57 ± 2.94 (42; 59)	0.0648
	**A20**	41 ± 2.12 (39.5; 44.5)	41 ± 1.83 (37; 46)	0.8096	39.5 ± 3.57 (33.5; 45.5)	59 ± 2.85 (45; 61)	0.0648
	**A25**	42 ± 1.73 (41; 45)	42 ± 1.78 (39; 48)	0.8854	41 ± 3.47 (35.5; 47)	60 ± 2.77 (46; 61)	0.0805
	**MCF**	44 ± 1.84 (42; 46.5)	43 ± 2.98 (40; 50)	0.9613	43.5 ± 2.92 (39; 49)	61 ± 2.62 (47; 62)	0.1199
	**ML**	10 ± 0.26 (10; 10)	10 ± 0.145 (10; 10)	0.6185	12 ± 0.84 (8; 12)	12 ± 0.39 (10; 12)	0.9558
**FIBTEM**	**CT**	69.5 ± 22.75 (55.5; 83.5)	50.8± 14.31 (40; 61)	0.0124	50 ± 7.71 (40.5; 63.5)	43.5 ± 3.13 (40; 50)	0.6167
	**A10**	26.5 ± 5.01 (22; 34.5)	25 ± 1.4 (19; 31)	0.7363	22 ± 3.12 (17; 25.5)	21 ± 1.43 (17.5; 27)	0.9635
	**A20**	28.5 ± 5.18 (23.5; 36.5)	27 ± 1.86 (20; 33)	0.6646	23 ± 3.5 (18; 27.5)	23 ± 1.53 (18.5; 29)	0.9634
	**MCF**	30 ± 5.57 (24; 38.5)	30 ± 3.99 (21; 37)	0.9613	23.5 ± 3.33 (18.5; 28)	23.5 ± 1.57 (18.5; 29.5)	0.9635
**APTEM**	**CT**	60 ± 21.67 (25; 100)	53 ± 5.12 (47; 58)	0.8000	54.5 ± 9.78 (47.5; 75)	56.5 ± 3.03 (44; 59.5)	0.3352
	**A10**	105 ± 36.04 (67; 189)	129 ± 21.29 (81; 181)	0.8000	151 ± 58.08 (130; 263.5)	119 ± 31.47 (73; 193)	0.2902
	**A20**	78 ± 0.88 (77; 80)	77 ± 2.84 (75; 78)	0.3642	80 ± 5.5 (69; 80)	77.5 ± 2.09 (70.5; 80)	0.7505
	**MCF**	44 ± 3.61 (41; 53)	45 ± 3.52 (40; 53)	0.9999	43.5 ± 2.69 (40; 48.5)	54 ± 2.75 (44.5; 62)	0.1482
	**ML**	10 ± 0.55 (10; 10)	10 ± 0.11 (10; 10)	0.7821	10 ± 0.42 (10; 11)	10 ± 0.15 (10; 10)	0.0722

Abbreviations: CT - clotting time [s], CFT - clot formation time [s], alfa-angle - steepness of curve [degree (o)], MCF - maximum clot firmness [mm], ML – maximum lysis [%], A10-A25 - firmness at time 10–25 minutes [mm].

Data are expressed as median ± standard error (interquartile range) - Me ± SE (Q1;Q3) *p* value ≥ 0.05 – not significant.

*p* value < 0.05 – statistically significant.

To sum up, these results suggest that the SOS/VOD (+) patients had a tendency to delayed activation of haemostasis with prolonged initial clot formation in the INTEM, EXTEM and FIBTEM assays.

### Parameters concerning clot firmness (MCF, A10, A15, A20, A25)

Analyses of the parameters concerning clot firmness, including maximum clot firmness (MCF), amplitude at 10, 15, 20 and 25 minutes (A10, A15, A20 and A25), did not differ between the patients with or without SOS/VOD on day +12 (Table 4). In summary, in our study the parameters concerning clot strength were unsuitable for the identification of SOS/VOD on day +12 in patients undergoing HSCT.

On day +28, INTEM-A10 and INTEM-A15 were significantly different between SOS/VOD (+) and SOS/VOD (−) indicating decreased clot firmness in the SOS/VOD (+) group. Other parameters related to the initiation of clotting (CT), fibrin polymerisation (CFT), stabilisation of the clot by fibrin and thrombocytes (MCF) and stability of the clot (ML) in all analysed assays did not differ statistically between the patients with or without SOS/VOD on day +28.

#### Parameters concerning clot lysis (ML, LI30, LI45 and LI60)

No differences were found in the parameters concerning clot lysis, including the maximum lysis and the lysis indices at 30, 45 and 60 minutes, between the SOS/VOD (+) and SOS/VOD (−) group. Therefore, the values of parameters concerning clot lysis did not assist in distinguishing the SOS/VOD (+) group from the patients without SOS/VOD symptoms.

#### Parameters concerning velocity profile (MaxVel, t-MaxVel and AUC)

The values of maximum velocity (MaxVel) did not differ in the SOS/VOD (+) group from those in the SOS/VOD (−) group. Except for a longer time to maximum velocity (t-MaxVel) in EXTEM on day +12 in the SOS/VOD (+) patients, no significant differences were found in results of t-MaxVel. Moreover, no differences in the area under the 1st derivative curve (AUC) were found between the analysed groups.

To summarise, our findings suggest that the velocity parameters were not able to differentiate between patients with or without SOS/VOD symptoms. Detailed data concerning the thromboelastometry values in patients with or without SOS/VOD are shown in Table 5.

**Table 5:** Comparison of coagulation dynamic properties analysed by ROTEM between the SOS/VOD (+) group and the SOS/VOD (−) group

ROTEM data		SOS/VOD (+)	SOS/VOD (−)	*p* value	SOS/VOD (+)	SOS/VOD (−)	*p* value
		Me ± SE (Q1; Q3)			Me ± SE (Q1; Q3)		
		day +12 after transplantation			day +28 after transplantation		
**INTEM**	**AUC**	4341.05 ± 138.34 (4258; 4354)	4341.05 ± 138.91 (3919; 4547)	0.6646	4946 ± 71.4 (3812; 7636)	5914.5 ± 2794.41 (4619; 6489)	0.0805
	**t-MaxV**	208.4 ± 14.8 (192; 246)	205.7 ± 7.88 (182; 209)	0.8854	198 ± 11.4 (198; 204)	193 ± 6.38 (178; 202)	0.2158
	**MaxV**	21.47 ± 0.96 (19; 24)	21.47 ± 1.19 (19; 22)	0.7363	20.0 ± 1.98 (17; 20)	19.5 ± 1.13 (16; 22)	0.8421
**EXTEM**	**AUC**	4428.47 ± 94.11 (4347; 4428)	4428.47 ± 129.21 (4190; 4554)	0.9613	4926 ± 225.32 (3837; 5140)	5140.85 ± 5140.86 (4644; 6043)	0.1200
	**t-MaxV**	68.0 ± 5.01 (66; 91)	61.0 ± 2.42 (55; 66)	0.0139	64.2 ± 3.96 (61; 67)	64.2 ± 64.24 (55; 64)	0.4618
	**MaxV**	23.16 ± 1.08 (22; 26)	23.15 ± 1.05 (19; 25)	0.7363	19.76 ± 1.98 (19; 22)	19.76 ± 19.76 (16; 22)	0.8291
**FIBTEM**	**AUC**	2952.84 ± 302.39 (2757; 3126)	2952.84 ± 149.21 (2556; 3404)	0.8855	2320.35 ± 185.05 (2136; 2512)	2320.35 ± 123.38 (2008.5; 2640)	0.9635
	**t-MaxV**	64.9 ± 7.72 (64; 86)	64.9 ± 3.52 (47; 66)	0.0624	56.3 ± 4.25 (55; 64)	53.5 ± 2.57 (49; 56)	0.5536
	**MaxV**	28.16 ± 2.79 (24; 28)	28.15 ± 1.15 (25; 31)	0.6646	26.05 ± 2.1 (26; 27)	26.03 ± 2.04 (22; 30)	0.8916
**APTEM**	**AUC**	6798.24 ± 476.45 (4434; 6798)	5649 ± 1721.97 (4455; 6798)	0.6765	4842.5 ± 154.56 (4033; 4842)	4842.5 ± 195.41 (4561; 5535)	0.2902
	**t-MaxV**	69.5 ± 6.46 (69; 79)	68.23 ± 5.52 (57; 69)	0.0676	63.7 ± 4.89 (61; 67)	62 ± 3.06 (53; 65)	0.5536
	**MaxV**	23.53 ± 2.29 (23; 24)	23.52 ± 1.12 (20; 26)	0.4323	19.25 ± 2.26 (19; 25)	19.25 ± 1.11 (14; 23)	0.2902

Abbreviations: MaxVel - maximum velocity [mm/min], MaxVel-t - time to maximum velocity [s], AUC – area under 1st derivative curve [mm × 100].

Data are expressed as median ± standard error (interquartile range) - Me ± SE (Q1;Q3) *p* value ≥ 0.05 – not significant.

*p* value < 0.05 – statistically significant.

### Cut-off values of CT for the prediction of SOS/VOD

**Figure 2:**
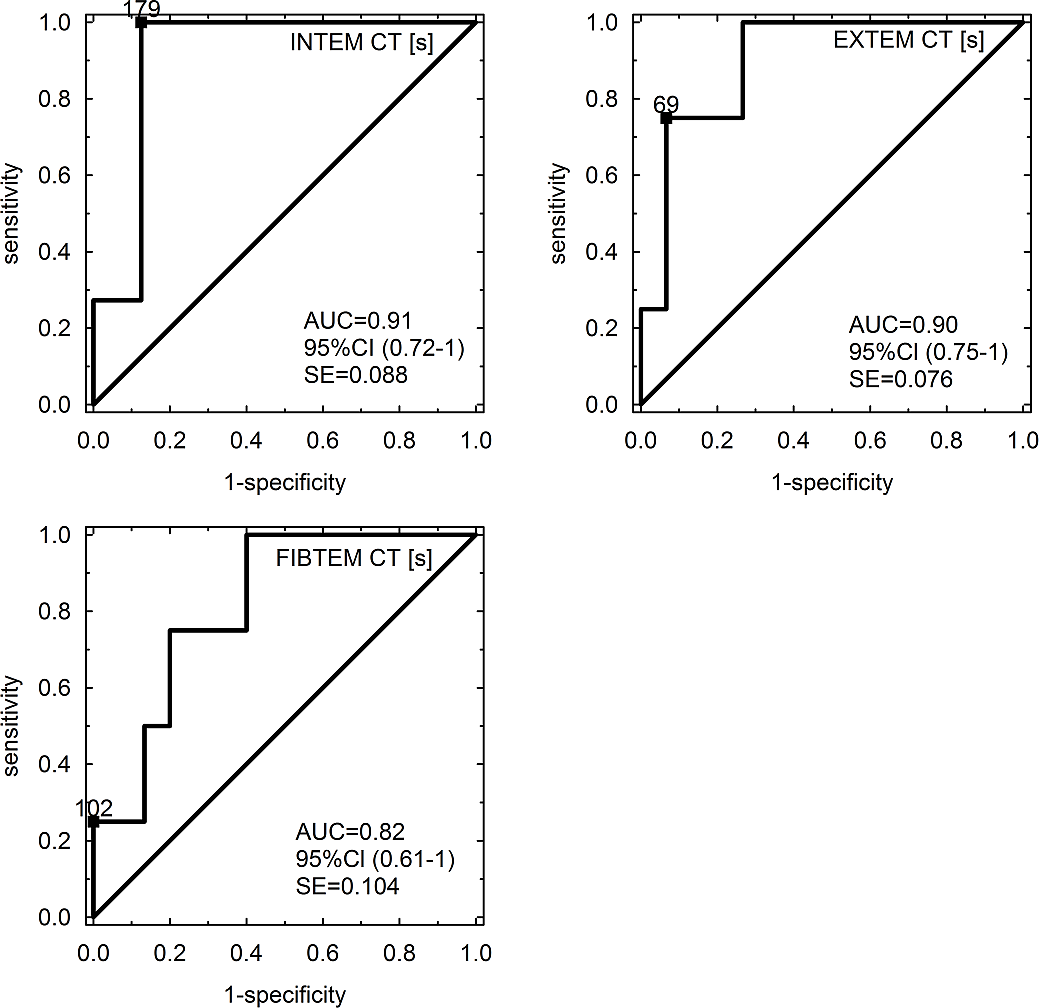
ROC curves of INTEM-CT, EXTEM-CT and FIBTEM-CT for SOS/VOD prediction Abbreviations: CT – clotting time [s]; AUC – area under the curve; CI – confidence interval; SE – standard error.

ROC analysis indicated a cut-off value of 179 s in INTEM-CT (AUC = 0.91; 95% CI 0.72–1.0, SE = 0.088), 69 s in EXTEM-CT (AUC = 0.90; 95% CI 0.75–1.0, SE = 0.076) and 102 s in FIBTEM-CT (AUC = 0.82; 95% CI 0.61–1.0, SE = 0.104) for the prediction of SOS/VOD in patients undergoing HSCT on day +12. ROC curves of INTEM-CT, EXTEM-CT and FIBTEM-CT for SOS/VOD prediction are provided in Figure 2.

### Other complications in the early post-transplant period

None of the patients developed a bleeding episode considered life threatening. In one patient, central catheter-related thrombosis was documented on day +5 after transplantation.

Gram-negative blood-stream infections (GNBSI), including *Klebsiella pneumonia*, *Enterobacter cloacae complex*, *Enterobacter aerogenes* and *Citobacter koseri* were found in four patients. The small sample size did not allow for thromboelastometry subanalyses.

## DISCUSSION

To our knowledge, this is the first analysis of haemostasis abnormalities in patients undergoing allogeneic HSCT for acute leukemia by rotation thromboelastometry together with an assessment of coagulation dynamic properties with respect to the occurrence of SOS/VOD.

The pathogenesis of SOS/VOD is complex, including endothelial injury and increased expression of adhesion molecules, pro-inflammatory cytokines and procoagulant factors [23–30]. A procoagulant state with a decreased level of antithrombin and protein C, consumption of factor VII, and an increased level of plasminogen activator inhibitor-1 (PAI-1) has been demonstrated at the time of SOS/VOD diagnosis in many studies [10–12, 31–34]. The level of PAI-1 is also important as a prognostic marker [10, 23, 35–38]. Studies are on-going including a quantitative mass spectrometry-based proteomics approach to identify candidate biomarkers of SOS/VOD [8]. Most of the biomarkers which have been reported so far as candidate clinical markers for SOS/VOD are not readily available or have only been examined in a limited number of patients making the results inconclusive.

Rotational thromboelastometry (ROTEM) enables the measurement of global clot formation and dissolution in whole blood in real time [39]. In whole blood, the interactions of coagulation factors, platelets, and fibrinogen with coagulation inhibitors during clot formation and subsequent fibrinolysis are analysed by ROTEM. The primary clinical application of ROTEM assays is in the assessment of haemostasis abnormalities to treat bleeding patients [39] as recommended in transfusion algorithms. Other studies indicate that perioperative ROTEM assays may detect patients at risk for postoperative thromboembolic complications [40]. As ROTEM assays allow for the assessment of both bleeding and thrombotic conditions, we evaluated their application in patients undergoing HSCT for acute leukaemia. So far, no information is available on the utility of thromboelastometry in the identification of patients at risk of SOS/VOD development. In addition, no data on the value of thromboelastometry in the management of post-transplant complications is available.

Our study provides new insights into the complex haemostasis abnormalities involved in the pathogenesis of SOS/VOD by the use of thromboelastometry. The preliminary results of our ROTEM analyses indicated that a delay in the activation of coagulation initiated by intrinsic and extrinsic activators, fibrin formation and fibrin polymerisation, independent of platelets in INTEM, EXTEM and FIBTEM assay on day +12, could be used to predict the occurrence of SOS/VOD in patients with acute leukaemia undergoing allogeneic HSCT. When compared to patients without SOS/VOD, patients with SOS/VOD had higher CT values in INTEM, EXTEM and FIBTEM assay, all suggesting impairment of fibrin polymerisation leading to abnormal clot structure and subsequently some defects in fibrinolysis. Complex haemostasis abnormalities including decreased factor VII activity, low thrombocytes count, abnormal expression of adhesion molecules, cytokines and procoagulant factors, together with the inhibition of anticoagulation pathway with decreased activity of antithrombin and protein C, and the concurrent inhibition of fibrinolysis by the increased level of PAI-1, all together may contribute to the impairment of thrombin formation and may influence the results of the whole blood thromboelastometry. In our study group, other ROTEM parameters concerning the speed at which a solid clot forms (CFT), clot firmness (MCF) and clot lysis (ML), together with velocity profile, were unable to differentiate between patients with, or without, SOS/VOD symptoms on day +12. This may be due to complex haemostasis impairment and the limited number of participants. However, on day +28 abnormalities in INTEM assay indicating reduced clot firmness in SOS/VOD (+) group were found. Moreover, both lower median values of MCF (reflecting clot firmness) and longer median CFT values (impairment of fibrin polymerisation) in INTEM and EXTEM assays indicates a trend to increased bleeding in patients with SOS/VOD on day +28.

Mild SOS/VOD is self-limiting and does not require specific treatment. Therapies for SOS/VOD of moderate severity are mostly supportive, with diuretics and fluid restriction, as was done in our cohort. The only agent approved for the treatment of severe SOS/VOD is defibrotide [6]. Defibrotide which, among other functions, stabilises endothelial cells by reducing endothelial-cell activation and damage, also reduces plasma levels of PAI-1 and results in the restoration of the thrombo-fibrynolitic balance [36, 38]. It has been shown that not only delay in the initiation of defibrotide has been associated with worse outcome [41, 42], but also early intervention is justifiable and effective [14, 38]. Therefore, early diagnosis and treatment of selected patients of mild to moderate SOS/VOD is essential to prevent disease progression and the development of severe SOS/VOD, and may possibly reduce mortality [6]. Defibrotide was not used on our patients, however, it is a treatment of first choice. Our findings regarding the thromboelastometry profile may allow for the determining of the optimal timing of the treatment of these patients but further studies are required.

The management of complex haemostasis abnormalities after HSCT is a challenge, not only because the patients are at increased risk of bleeding and thrombotic complications, but also because conventional coagulation tests may be unsuitable in the assessment of haemostasis. APTT and PT, did not differ significantly between our patients with, or without, SOS/VOD in the present study. This observation is in line with other studies evaluating the effects of defibotide on SOS/VOD [15, 45], in which most of the patients showed normal ranges of PT, APTT and fibrinogen but contrary to the results of Sartori et al. [43]. In the light of these data, it is possible that not only may ROTEM assessment be useful in the prediction of SOS/VOD occurrence but may also be appropriate to monitor the treatment with defibrotide or other agents (for example shortening of CT in ROTEM assays) or may assist in determining the optimal management of patients with SOS/VOD.

Although all our patients were at high risk of developing SOS/VOD, the incidence reached 26% in our study cohort (7/27), including mild disease - an incidence which is still within the range described in literature. The high rate of SOS/VOD documented in the early phases of HSCT was related to the high number of patients who had one or more conditions associated with a significant increased risk of developing SOS/VOD. Identification of the risk factors associated for the development of SOS/VOD is essential for adequate monitoring and early intervention and therapy [6]. Our data show that patients with pre-transplant ferritin levels above 1000 ng/dl had an increased risk of SOS/VOD development which is consistent with literature [45–47]. Due to the limited number of patients studied, we were not able to demonstrate that other recently described patient-related or transplant-related factors had an impact on SOS/VOD occurrence. Most of the identified risk factors were found in either paediatric or adult patient populations, which were sometimes heterogeneous with regard to underlying disease and chemotherapeutic conditioning regimens.

Our study has some limitations. The number of participants was small, none of our patients had episodes of life–threatening bleeding or thrombotic events during the study and none required surgical procedures.

In conclusion, the baseline characteristics of haemostasis defects, as determined by thromboelastometry in whole blood, may be important in understanding the pathophysiology involved in SOS/VOD development by identification of specific biomarkers in ROTEM assay, indicative of SOS/ VOD occurrence. Our preliminary results provide new insights into the haemostasis abnormalities in adult patients undergoing HSCT for acute leukemia, with regard to SOS/VOD symptoms. In the SOS/VOD (+) patients we have demonstrated a significant delay in the initiation of thrombin formation in the analysed ROTEM assays. The question of whether our findings may assist in the better management of patients undergoing HSCT should be clarified in a further prospective study.

## MATERIALS AND METHODS

### Patients

Twenty-seven adult with acute leukaemia, qualified for allo-HSCT between June 2011 and June 2012, were included in the study.

The diagnosis of SOS/VOD was established using the Baltimore criteria (total bilirubin ≥ 2.0 mg/dL with ≥ 2 of the following: hepatomegaly, ascites or 5% weight gain. Doppler ultrasound was performed to confirm clinical findings (hepatomegaly and ascites) and for differential diagnoses [14]. The clinical evaluation of SOS/VOD severity was performed retrospectively based on measurable clinical data including assessment of the rate of changes in the level of bilirubin, liver and renal function tests, and the amount and pace of weight gain above baseline, as proposed by Chao, with Carreras modification (Table 6) [5–7]. Clinical and laboratory assessment during hospital stay was performed daily.

**Table 6:** Grade of SOS severity [5–7]

Clinical data	SOS Grade		
	Mild*	Moderate*	Severe*
Bilirubin (mmol/l)	34.2–51.3	53–85.5	> 85.5
Liver function tests^*^	< 3 × normal	3–5 × normal	>5 × normal
Weight above baseline	2%	2.1–5%	>5%
Renal function^***^	Normal	< 2 × normal	≥ 2 × normal
Rate of change, days	Slow (over 6–7 days)	Moderate (over 4–5 days)	Rapid (over 2–3 days)

^*^Where two or more of the listed parameters are found.

^*^Serum aminotransferases.

^***^Serum creatinine.

Resolution of SOS/VOD was defined as normalization of serum bilirubin, resolution of ascites, and return of body weight to baseline values [15]. Based on the published data related to transplant- and/or patient- and disease-related factors for the development of SOS/VOD after HSCT, the following data were analysed: age, recipient gender, type of leukaemia and its stage, myeloablative conditioning (busulfan- or total body irradiation (TBI)-based), alternative donor, iron overload (pre-transplant ferritin level above 1000 ng/dl), previous HSCT and liver disease [1, 5, 6].

Neutrophil engraftment was defined as an increase in an absolute neutrophil count greater than, or equal to, 0.5 G/l for the first three consecutive days post-HSCT. Acute GvHD was diagnosed according to the IBMTR criteria [16, 17] and diagnosis of chronic GvHD was based on the NIH consensus criteria [18]. Organ toxicity was assessed according to the Common Terminology Criteria for Adverse Events (CTCAE). A serious bleeding event was defined as one that was life-threatening or resulted in prolonged hospitalization and occurring within 30 days after HSCT [19]. Bloodstream infection was defined as the isolation of a bacterial pathogen from at least one blood culture [20]. Advanced disease was defined as acute leukaemia beyond second complete remission (CR) or disease relapse.

### Thromboelastometry

Fasting blood samples were collected for coagulation testing into vacuum tubes with minimal stasis (2.9 ml S-Monovette^®^ Coagulation 9NC/3 ml, 3.2% Sodium Citrate, Sarstedt, Nümbrecht, Germany). Rotation thromboelastometry was performed by a ROTEM^®^ coagulation analyser according to the manufacturer's instructions (ROTEM^®^ Gamma, Pentapharm, Munich, Germany) as previously described [21]. Four standard ROTEM assays, including INTEM, EXTEM, FIBTEM and APTEM were conducted before the conditioning regimen start (day -10), on the day of stem cell infusion (day 0) and on day +12 and +28 after HSCT. The INTEM and EXTEM assays represent the contact system (or intrinsic coagulation pathway), and tissue factor activation (or extrinsic coagulation pathway), respectively. FIBTEM allows for the qualitative analysis of the fibrinogen levels and fibrin polymerisation independent of platelets, and is performed as EXTEM with inhibition of platelets by cytochalasin D. The APTEM assay is carried out to evaluate fibrinolysis using a fibrinolysis inhibitor (aprotinin). The following parameters were assessed: clotting time (CT), clot formation time (CFT), α-angle, maximum clot firmness (MCF), amplitude at 10, 15, 20 and 25 minutes (A10, A15, A20, A25), maximum lysis (ML) and lysis index at 30 min (LI 30), 45 min (LI 45) and 60 min (LI 60). Assessment of the coagulation dynamic properties was performed as the first derivative of the thromboelastometry curves (velocity profile), as described by Sorensen et al. [22]. For all tests, the recorded ROTEM parameters of velocity profile were: MaxVel, t-MaxVel and the area under the velocity curve (AUC). All coagulation tests and ROTEM tests were performed within 30 minutes of sample collection.

To find the influence of platelets, fibrinogen and routine coagulation tests (activated partial thromboplastin time and protrombin time) on the ROTEM parameters, a full blood count with platelet count (× 10^9^/l), routine coagulation tests and plasma fibrinogen were determined by routine laboratory techniques (Instrumentation Laboratory Company - Bedford USA).

The Bioethical Committee of Poznan University of Medical Sciences approved the study and the patients provided their written informed consent, in accordance with the Declaration of Helsinki.

### Statistical methods

The results are presented using methods of descriptive statistics such as frequency (n), medians and standard error (SE) or constant with interquartile ranges (IQRs: 25%–75%) and non-outlier. The Shapiro-Wilk test was performed to assess normality. In order to compare differences between the groups, the chi-square test or Fisher's exact test was used for categorical variables and the Mann-Whitney U test for continuous variables.

The ability of each single ROTEM parameter to discriminate between patients with, or without, SOS/VOD was also evaluated with receiver operating characteristic (ROC) curve analysis. For each parameter, the area under the ROC curve (AUC) was identified to determine the sensitivity and specificity with a 95% confidence interval (CIs) at cutoff values for the analysed parameter predictive of SOS/VOD occurrence. A *p-value* below 0.05 was regarded as statistically significant. The statistical analyses were performed with STATISTICA 10 and STATISTICA Medical Package 2.0 (StatSoft, Inc. 2012 software, Tulusa, USA).
